# Biotite: new tools for a versatile Python bioinformatics library

**DOI:** 10.1186/s12859-023-05345-6

**Published:** 2023-06-05

**Authors:** Patrick Kunzmann, Tom David Müller, Maximilian Greil, Jan Hendrik Krumbach, Jacob Marcel Anter, Daniel Bauer, Faisal Islam, Kay Hamacher

**Affiliations:** 1 Computational Biology and Simulation, Technical University of Darmstadt, Schnittspahnstraße 2, 64287 Darmstadt, Germany; 2 Department of Computer Science, Eberhard Karls University of Tübingen, Sand 14, 72076 Tübingen, Germany; 3 Independent Researcher, Heidelberg, Germany

**Keywords:** Open source, Python, Structural bioinformatics, Sequence analysis

## Abstract

**Background:**

Biotite is a program library for sequence and structural bioinformatics written for the Python programming language. It implements widely used computational methods into a consistent and accessible package. This allows for easy combination of various data analysis, modeling and simulation methods.

**Results:**

This article presents major functionalities introduced into Biotite since its original publication. The fields of application are shown using concrete examples. We show that the computational performance of Biotite for bioinformatics tasks is comparable to individual, special purpose software systems specifically developed for the respective single task.

**Conclusions:**

The results show that Biotite can be used as program library to either answer specific bioinformatics questions and simultaneously allow the user to write entire, self-contained software applications with sufficient performance for general application.

**Supplementary Information:**

The online version contains supplementary material available at 10.1186/s12859-023-05345-6.

## Background

*Python* is a general purpose programming language that is popular for its easy usage and rapid development. However that ease of usage comes at the cost of computational speed: Due to *Python*’s code interpretation at runtime and its convenient features such as dynamic typing and garbage collection, the execution requires significant overhead compared to most compiled programming languages.

One way of mitigation is to run code written in *C* using a *pythonic* foreign-language interface. This feature has been harnessed by the *Numerical Python* (*NumPy*) package [1], which introduced *n*-dimensional arrays, or *ndarrays* in short, to store numerical data. Numerical operations on an *ndarray* are vectorized, i.e. they are applied to each of the array’s elements using underlying extension modules, which renders the computation speed on large datasets orders of magnitude faster than in pure *Python*.

The combination of the advantages of *Python* with these fast vectorized numerical operations has lead to an increasing attention by various areas of science: Today the *Python* scientific computing ecosystem comprises program libraries^1^ from quantum mechanics calculations [2] to astronomical applications [3].

The open-source package *Biotite* (https://www.biotite-python.org/) aims to fill this role for the realm of computational molecular biology. It provides editing and analysis tools for sequences and 3D molecular models. In contrast to other comparable bioinformatics libraries like *Biopython* [4], which originally dates back to the time prior to the release of *NumPy*, *Biotite* integrates *NumPy* arrays directly into its data model for sequences and structures. The vectorization substantially accelerates operations like geometric measurements on structure models or DNA sequence translation into protein. Where vectorization with *NumPy* is not applicable, *Biotite* employs extension modules written in *Cython* [5] to speed up time-consuming computations. If the user is accustomed to *NumPy*, handling objects in *Biotite* is intuitive: filtering for particular atoms in a structure or regions in a sequence accepts the same indexing semantics as *NumPy* and functions return *ndarrays* for pure numerical values.

Since its initial publication of *Biotite* [6], a multitude of new functionalities have been added. In this article we highlight the arguably most important additions of recent years.

### Package organization

*Biotite* comprises four subpackages: biotite.database contains functions to search in and fetch data from *RCSB PDB* [7], *NCBI Entrez* [8] and *UniProt KB* [9] via their REST APIs. biotite.sequence contains methods for reading, writing, editing and analyzing sequence data, whereas biotite.structure is the counterpart for structure data. To extend *Biotite* with analysis of external software biotite.application provides seamless interfaces to programs like *Clustal Omega* [10] or *DSSP* [11].

The *Biotite* project follows the paradigm, that only established methods in computational molecular biology are implemented in *Biotite*. Functionality that is tailored for rather uncommon tasks, uses novel algorithms or requires additional dependencies is therefore released as an *extension package*. The functionalities in these packages integrate tightly with the data model used by *Biotite*, but are developed and distributed independently.

### Data model

As already outlined, *Biotite* uses *ndarrays* to store data where possible. Hence, a Sequence object internally uses an *ndarray* to store its symbols. Although in the biological context the set of allowed symbols in the sequence, the *alphabet*, comprises typically ASCII characters representing nucleobases or amino acids, *Biotite* defines sequences in a broader sense, by allowing any object to be part of an alphabet. To make this decision compatible with the numerical nature of *ndarrays*, *Biotite* harnesses the fact, that most alphabets are relatively short: Each symbol is translated into a unambiguous integer, its *symbol code*, based on the position in the underlying alphabet. For example in the alphabet $\{A, C, G, T\}$, *A* would be translated into 0, *C* into 1, etc. This approach yields performance advantages in accessing substitution matrices and indexing *k*-mers. The *symbol codes* for each symbol in a sequence are stored in an internal *ndarray* of the Sequence.

A sequence alignment depicts which positions in one sequence correspond to positions in one or multiple other sequences. Alignment objects in *Biotite* fulfill this purpose. They store the Sequence objects corresponding to the aligned sequences and a *trace*: a 2-dimensional *ndarray*, where each row contains the respective sequence positions in the alignment column.

Macromolecular structures can be thought of as a list of atoms, where each atom is defined by its position and further annotations, like its name, its element, the residue it is part of, etc. The straightforward solution to represent structure data as such a list would impede proper vectorization with *NumPy*. Thus *Biotite* implements a structure model as collection of *ndarrays*, wrapped by an AtomArray object. An AtomArray contains an $(n \times 3)$-dimensional array for the coordinates of the *n* atoms and one *n*-dimensional array for each annotation. For multi-model structures, such as NMR models or trajectories from molecular dynamics simulations, an AtomArrayStack can be used, where the coordinates are $(m \times n \times 3)$-dimensional instead to account for *m* models.

## Implementation

### Alignment searches

**Fig. 1:**
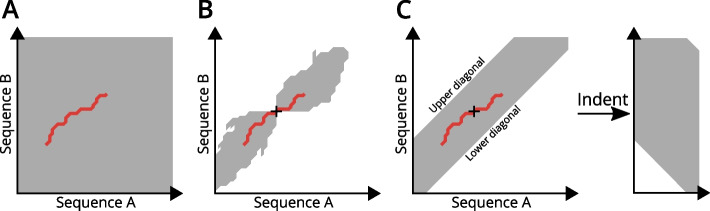
**Gapped sequence alignment methods.** Each plot shows a schematic dynamic programming table for a local alignment method. The gray area depicts the explored portion table, i.e. the part that is actually computed. The ‘+’ marks a *k*-mer match position. The red line indicates the best alignment. **A** Rigorous sequence alignment. The complete table is explored. Hence no match position is required as seed. **B** Alignment with *X*-drop criterion. Exploration of the table is terminated at positions, where the alignment score drops *X* below the score of the best alignment seen so far. In consequence, the shape of the explored area is dependent on the sequences. **C** Banded alignment. Table exploration is restricted to a diagonal band, i.e. only a certain number of gaps is allowed in either sequence. In *Biotite* the dynamic programming table is indented to reduce memory requirements by removing a large part of the unexplored area

With the release of *BLAST* [12], *k*-mer based alignment searches became the prevalent method for rapid identification of homologs in a sequence database. In modern software such as DIAMOND [13] or *MMseqs2* [14], alignment searches are a multi-stage process: In each stage a number of alignment candidates are filtered out, reducing the run time massively in the later more time consuming and sensitive stages. *Biotite* maps these stages to separate functions and objects, forming a modular toolkit for alignment searches: The user can choose between different alternatives of methods for each stage, and can optionally introduce a custom implementation for parts of the alignment search.

In the beginning of a typical workflow, the *k*-mers of each sequence in a database, i.e. all contiguous subsequences of length *k* are indexed into a table that maps each *k*-mer to the sequence positions where it appears. For this purpose *Biotite* provides the KmerTable class, which uses an internal *ndarray* of *C*-arrays for mapping *k*-mers to positions.

Each *k*-mer is unambiguously mapped into a code $d = \sum _{i=0}^{k-1} q^i c_i$, using it symbol codes $c_i$. *q* is the length of the sequence alphabet. This mapping is performed for each *k*-mer in a sequence, resulting in another sequence containing the values for *d*. Spaced *k*-mers including ‘*don’t care*’ positions [15] can be used here as alternative to continuous ones. A KmerTable is created in two passes [14]: In the first pass the *ndarray* counts the number of occurrences of each possible *d*, resulting in $q^k$ elements. In the second pass, the *ndarray* replaces each count with a pointer to a new *C*-array containing the sequence positions. As the size of each *C*-array is known from the first pass, time consuming array resizing is prevented. In addition, the ability of a KmerTable to be combined from multiple KmerTable instances and to be serialized, makes the class suitable for multiprocessing on multiple cores.

To prevent the appearance of spurious homologies, low-complexity regions can be masked in the KmerTable creation. Low-complexity regions are typically identified using the *tantan* program [16], interfaced in the TantanApp for convenience.

In the first stage of an alignment search, matching *k*-mer positions between the database and the query sequence are found by lookup in the KmerTable. The result is a $(n \times 3)$-dimensional *ndarray* containing the *n* matches as tuple of query sequence position, database sequence id and database sequence position. To find matches of similar *k*-mers instead of strictly equal ones, a substitution score threshold can be given to relax the matching condition [17]. The *ndarray* of matches may be subjected to further custom filtering, such as a *two-hit* strategy [17], before it is used in downstream stages.

The fast *k*-mer matching is usually followed by an ungapped alignment stage and finally a gapped alignment stage, where each remaining match position is used as alignment seed. Local ungapped seed extensions [12] are performed with align_local_ungapped(). In addition to the existing slow rigorous method [18, 19] (align_optimal(), Fig. 1A), *Biotite* now also offers the *X*-drop [12, 20] (align_local_gapped(), Fig. 1B) and band heuristics [21] (align_banded(), Fig. 1C) for gapped alignments. Due to the typically smaller alignment search space their computation time is drastically reduced compared to the rigorous approach.

For statistical assessment of the obtained alignment scores, their E-value [22] can be calculated using the EValueEstimator class. At initialization, the EValueEstimator object samples a large number of alignment scores from randomized sequences and uses the method of moments [23] to estimate the parameters of the score distribution.

### Trees and multiple alignments

Although the *multiple sequence alignment* (MSA) programs interfaced in the application subpackage [10, 24, 25] are sufficient for most applications, their flexibility with respect to scoring schemes and supported sequence alphabets is limited and they are usually only available on *Unix*-based operating systems. As alternative *Biotite* offers the align_multiple() function, that implements the simple original progressive alignment algorithm [26] to align given Sequence objects, based on customizable substitution matrix and gap penalty.

The progressive alignment procedure requires a guide tree, that determines in which order the sequences are aligned. By default, the guide tree is created using the UPGMA hierarchical clustering method from pairwise sequence distances [27]. However, the tree can alternatively be created using any other method or read from *Newick* notation. Besides UPGMA which is available as upgma() function, *Biotite* provides the *neighbor-joining* method [28] (neighbor_joining()) as well.

### Sequence profiles

*Biotite* is able to create sequence profiles from multiple sequence alignments consisting of nucleotide, protein or custom sequences. The usefulness of profiles lies in their better representation of information than a consensus sequence or a multiple sequence alignment [29].

In the literature, there are a lot of ambiguous terms describing the same matrices used: a sequence profile can be either represented as a *Position Frequency Matrix* (PFM), a *Position Probability Matrix* (PPM) or a *Probability Weight Matrix* (PWM) [30]. In a PFM, for each position the total count *C* of each symbol *S* in the used alphabet is stored. In a PPM, the probability *P* of each *S* in the PFM is calculated for each position as

$$\begin{aligned} P(S) = \frac{C_S + \frac{c_p}{k}}{\sum _{i} C_i + c_p}. \end{aligned}$$

$c_p$ denotes optional pseudocounts and *k* the number of symbols in the alphabet. In a PWM, for each position a log-odds score *W* is assigned to every symbol with

$$\begin{aligned} W(S) = \log _2 \left( \frac{P(S)}{B_S} \right) , \end{aligned}$$

where *B* denotes background frequencies.

The SequenceProfile class stores information about a sequence profile of aligned sequences. This class saves a PFM of the occurrences of each alphabet symbol at each position using a $(n \times k)$-dimensional *ndarray*, where *n* is the sequence length of the aligned sequences and *k* is the number of symbols in the alphabet. It also saves the number of gaps at each position in an *ndarray* with length *n*. The PFM, the gaps and the alphabet used in a SequenceProfile object are directly accessible attributes of the class. from_alignment() can be used to create a SequenceProfile object from an indefinite number of aligned sequences.

The method to_consensus() gives the consensus sequence of a SequenceProfile object. In case there is more than one symbol with maximum occurrences in a profile position, nucleotide sequence profiles use IUPAC ambiguity symbols. For other sequence types, the first symbol with maximum count in the alphabet is chosen.

probability_matrix() gives the PPM of the profile with optional pseudocount. This is used for sequence_probability() and sequence_score(). With the first method, the sequence probability can be calculated. The sequence probability is the product of the probability of the respective symbol over all sequence positions. With the second one the score for a sequence can be calculated: The score is the sum of weights *W* of the respective symbol over all sequence positions.

### Unit cells and macromolecular assemblies

AtomArray objects have a box attribute, a $(3 \times 3)$-*ndarray* that represents the vectors of the unit cell or, in the context of an molecular dynamics simulation, the simulation box. For an AtomArrayStack the box accommodates a $(m \times 3 \times 3)$-dimensional *ndarray*, since each model may have a different box. The box is automatically read from structure file, if available, and can be used for geometric measurements, that take periodic boundary conditions into account. Furthermore, a number of box-related functions is available, for example to reassemble chains separated by periodic boundaries (remove_pbc()) or to add periodic copies to the structure (repeat_box()).

Macromolecular assemblies represent the putative functional form of a protein (complex). However, the atom coordinates in structure files from for example the PDB are related to the experiment: For instance, in X-ray crystal structures the coordinates describe the asymmetric unit of the protein crystal, but not necessarily the active conformation of a complex. *PDBx/mmCIF* files provide instructions, how the coordinates of molecular chains need to be copied and transformed, to obtain a certain macromolecular assembly. This information can be read from *PDBx/mmCIF* format by get_assembly(), returning an AtomArray representing the respective assembly.

### Partial charges

The introduction of the partial_charges() command extended *Biotite*’s capabilities with the computation of partial charges of various molecule classes, ranging from biological macromolecules such as proteins and nucleic acids to small molecules such as ligands. The function represents an implementation of the *Partial Equalization of Orbital Electronegativity* (PEOE) algorithm [31]. Partial charge computation is based on the formal atom charges associated with the input AtomArray and relies on an array of tabulated parameters originating from the original publication. Hence, it is restricted to those elements and valence states for which parameters are available. However, the tabulated parameters comprise most elements relevant in the biochemical context, including halogens which may occur in ligands. As the underlying algorithm is iterative, the amount of iterations can be chosen by the user depending on the desired precision of the result. The code is written in Cython [5], to achieve fast computation.

### Small molecules

While previously *Biotite* focused on macromolecular structures, the support for small molecules improved in recent releases. With the MOLFile class, small molecule structures can be read from *MOL* as well as *SDF* files [32]. Using information from the *chemical components dictionary* (CCD) [33], an AtomArray representing a desired small molecule can be also created from scratch given merely the residue name using the residue() function. The entire catalog of small molecules from the CCD is available here, comprising all molecules that are part of any PDB entry. Furthermore, *Biotite* provides an interface to *AutoDock Vina* [34] (VinaApp), that allows molecular docking of small molecules to proteins. By using its own PDBQTFile reader/writer and partial charges from PEOE, *Biotite* does not require an additional installation of *AutoDockTools* [35] to prepare input for *Vina* and to parse its output.

### Bond prediction

AtomArray and AtomArrayStack objects are able to store atom connectivity including the bond order. Bonding information can be read from *PDB* and *MMTF* [36] files. Alternatively, structures can be annotated with bonding information afterwards: If the residues in the structure model are comprised by the CCD, bonds can be automatically determined by connect_via_residue_names(). Otherwise, connect_via_distances() is available as fallback, which assumes bonds for all pairs of atoms, whose distance is within the bounds of the known bond length for the respective combination of elements [37]. However, the bond order cannot be inferred this way.

### Hydrogen bonds

**Fig. 2:**
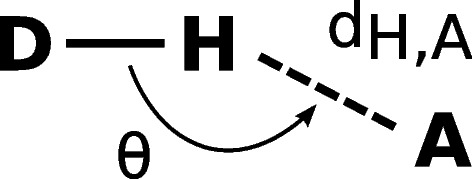
**Hydrogen bond detection method.** Hydrogen bonds are detected by an angle and distance criterion: The angle $\Theta$ is formed by the donor (D), a hydrogen atom (H$_D$) bound to D, and the acceptor (A) and the distance $d_{H,A}$ between atoms A and H

Hydrogen bond detection (hbond()) employs the Baker-Hubbard algorithm [38]. Possible interaction sites are identified based on the angle $\theta$ between donor (D), donor hydrogen (H$_D$) and acceptor (A) and the distance d$_{H,A}$ between H$_D$ and A (Fig. 2). By default, $\Theta \ge 120^\circ$ and $d_{H,A} \le 2.5$ Å, but values can be adjusted. For detection, hbond() only considers the heavy elements O, N, S. Bonding information is used to efficiently identify donor hydrogen atoms if available. Otherwise, possible hydrogens are identified by a distance cutoff ($d_{D,H} \le 1.5$ Å).

The algorithm returns a $N\times 3$ matrix containing triplets of atom indices for D, H$_D$ and A. When supplied with an AtomArrayStack, hbond() also returns a $M\times N$ mask indicating the presence of interaction $n_i$ in model $m_i$. A utility function hbond_frequency() to obtain hydrogen bonding frequencies from the mask is provided for convenience.

### Nucleic acid secondary structures

**Fig. 3:**
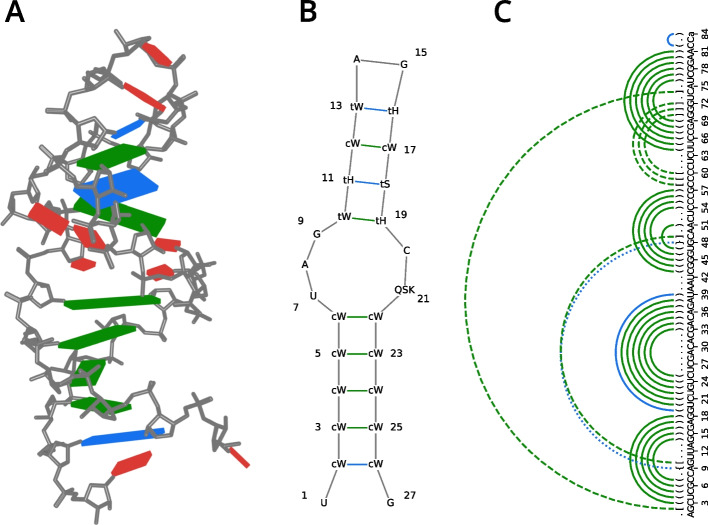
**Different visualizations of nucleic acid secondary structure features.** The atom coordinates and annotations of **A** and **B** are taken from PDB 6ZYB [39]. For **C** they are taken from PDB 4P5J [40]. Green lines/cuboids denote canonical base pairs, while blue lines/cuboids denote non-canonical base pairs. The base pairs were elucidated using base_pairs(). **A** Visualization of base pairing interactions with *Pymol*. Red cuboids indicate unpaired bases. **B** Visualization of base pairing interactions and their Leontis-Westhof annotations using plot_nucleotide_secondary_structure(). The first letter of the paired base annotations indicates the relative orientation of the glycosidic bonds (c - cis, t - trans), while the second letter encodes the interacting edges (S - Sugar, W - Watson-Crick, H - Hoogsteen/C-H). The relative glycosidic bond orientations and interacting edges were elucidated using base_pairs_glycosidic_bonds() and base_pairs_edge() respectively. **C** Visualization of base pairing interactions as an arc diagram created with *Matplotlib*. The dot-bracket-letter notation was generated with dot_bracket(). Solid arcs indicate pseudoknot order zero, dashed arcs pseudoknot order one, and dotted arcs pseudoknot order two. Capital letters represent canonical bases, while lowercase letters represent non-canonical bases mapped to the canonical base indicated by the respective one-letter-code. The bases were mapped using map_nucleotide()

*Biotite* contains a broad range of methods for nucleic acid secondary structure analysis which generally support canonical as well as non-canonical bases and base pairings.

Mapping of non-canonical to canonical bases allows for approximation of geometric information by superimposition of canonical structural features. For this purpose the function map_nucleotide() is introduced to the structure subpackage. Bases are superimposed based on matching *PDB atom names* and mapped to the canonical base with the lowest *RMSD*. Bases are only mapped if the given *PDB residue name* is classified as a DNA/RNA polymer according to the CCD, there is a match of at least three atom names, and the *RMSD* is below a threshold of 0.28 Å, recommended in the literature [41]. Given a residue, which is supplied as AtomArray, the function returns the base that the residue was mapped to and whether the mapping was an exact match, thus a canonical nucleotide. The minimum number of atoms necessary as well as the *RMSD* threshold are customizable.

The function base_stacking() allows for the detection of aromatic base stacking according to the following criteria [42]: Stacking is assumed if the distance between aromatic ring centers is $\le 4.5$ Å, the angle between the ring normal vectors is $\le 23^{\circ }$, and the angle between the distance vector connecting the ring centers and the normal vectors of both bases is $\le 40^{\circ }$.

The function base_pairs() detects base pairs according to the *DSSR* criteria [41]. A base pair is considered as present, if the distance between the base origins according to the standard reference frame [43] is $\le 15$ Å, the vertical separation between the base planes is $\le 2.5$ Å, the angle between the base normal vectors is $\le 65^{\circ }$, the bases do not exert stacking, and the bases are connected by at least one hydrogen bond. A visualization of the base pairs detected in a fragment of the *Sarcin-Rycin* loop of *E. coli* (PDB 6ZYB [39]) is shown in Fig. 3A.

Both base_stacking() and base_pairs() take the structure to be analyzed (an AtomArray) as input. base_pairs() also allows constraining interactions such that each base is only paired to one other base, preferring pairings with a higher number of hydrogen bonds. Both functions return a $N \times 2$ matrix, where each row corresponds to the first indices of the interacting bases in the input AtomArray.

The dot-bracket-letter notation [44] describes secondary structures unambiguously by assigning a pseudoknot order for nested sets of base pairs. The function pseudoknots() implements a dynamic programming algorithm [45], that determines all optimal solutions for assigning the pseudoknot order such that the number of base pairs is maximized at each level and decreases as the pseudoknot order increases. It is also possible to set a maximum pseudoknot order to speed up calculations.

The function dot_bracket() relies on pseudoknots() to generate all optimal dot-bracket-letter notations for a given sequence length and set of base pairs referencing positions in the sequence. The function dot_bracket_from_structure() generates all dot-bracket-letter notations directly from a given AtomArray. As an example the secondary structure of a *tRNA* mimic from the turnip yellow mosaic virus (PDB 4P5J [40]) visualized as an arc diagram using *Matplotlib* together with the corresponding dot-bracket-letter notation is shown in Fig. 3C.

The Leontis-Westhof Nomenclature [46] distinguishes pairs of canonical bases by the relative orientations of the glycosidic bonds (cis, trans) as well as the interacting edges (Sugar, Watson-Crick, Hoogsteen/C-H). The functions base_pairs_glycosidic_bonds() and base_pairs_edge() can be used to determine these properties, respectively, from a given AtomArray and base pairs. The relative orientation of the glycosidic bonds is calculated as suggested by Yang *et al.* [47].

Furthermore, the application subpackage was extended with interfaces to the programs *RNAfold*, *RNAalifold* and *RNAplot* of the *ViennaRNA* package [48]. The RNAfoldApp and RNAalifoldApp classes can be used to predict RNA secondary structures for a given sequence using *RNAfold* or for a given alignment using *RNAalifold*, respectively. Likewise, the RNAplotApp class can be used to generate coordinates for a 2D plot of a given secondary structure.

plot_nucleotide_secondary_structure() takes advantage of the interface to *RNAplot* to create highly customizable two dimensional plots from a given sequence and corresponding base pair matrix. Figure 3B was generated using this function. The sequence data at the positions of base pairings was augmented by the Leontis-Westhof nomenclature, which enables the representation of base orientation.

### Elastic network models

*Springcraft* (https://springcraft.biotite-python.org/) is the dedicated *Biotite* extension for normal mode analysis (NMA) with coarse-grained elastic network models (ENMs) of proteins. In ENMs, amino acid residues are abstracted as nodes, which are commonly assigned to positions of C$_{\alpha }$ atoms, while pairwise interactions between them are modeled as Hook’ean springs [49–51]. They allow to understand stabilization of a protein’s fold [52] as well as the inference of functionally important residues and representative structural elements [53–55].

Both gaussian network models (GNMs) and anisotropic network models (ANMs) are implemented in *Springcraft* [49, 56]. A varied selection of ENM force fields is available, ranging from the original parametrization of GNMs/ANMs with invariant force constants to distance- (pfENM, Hinsen-C$_{\alpha }$-parametrization) and amino acid sequence-dependent variants (sdENM, eANM) [57–60]. Force fields are represented as ForceField base class, with subclasses for the different preset force fields. Custom ENM force fields can be readily defined by inheriting and modifying the ForceField base class. A granular modification of single interactions for a given ForceField is possible with the PatchedForceField subclass: Single pair contacts can be established, shut off or assigned a specific force constant independently. This greatly improves upon previous methods to model specific interactions [61].

With an AtomArray and a ForceField object as input for the GNM/ANM classes, NMA is conducted with separate instance methods: It is possible to compute the covariance matrices, eigenvectors and commonly derived quantities, such as residue displacements, fluctuations for a given mode or dynamical cross-correlations between residues. Another method of the ANM class allows the prediction of structural changes in proteins upon ligand binding by applying linear response theory [62].

### Molecular visualization

To create publication-ready molecular visualizations AtomArray objects can be transferred to the popular *PyMOL* software suite [63] with the help of the *Ammolite* extension package (https://ammolite.biotite-python.org/). The structure migration uses the *Python* API of *PyMOL*, eliminating the need of intermediate structure files and the associated potential loss of information. For each transferred AtomArray, *Ammolite* creates a PyMOLObject that links the AtomArray to the newly created *PyMOL* object. Commands, like coloring atoms and changing representations, can be called from this PyMOLObject with the benefit, that *NumPy* based atom selections can be used as alternative to string-based *PyMOL* selection algebra. Finally, *Ammolite* provides convenience functions to create *compiled graphics objects*, facilitating the addition of 3D shapes, such as balls and cylinders, into molecular visualizations.

### Miscellaneous extension packages

Further extension packages have been published in recent years: *Gecos* (https://gecos.biotite-python.org/) [64] is a software for generating optimal color schemes for sequence alignment visualizations on the basis of weight or substitution matrices.

*Hydride* (https://hydride.biotite-python.org/) [65] is a package to predict hydrogen positions for those structure models, in which hydrogen atoms could not resolved experimentally. This enables hydrogen bond measurement and accurate base pair identification on a wider range of structures.

## Results and discussion

### Example application

**Table 1:** Top 10 of identified sequences homologous to human $\alpha$-globin

Rank	Gene	ID	E-value	Identity (%)	Coverage (%)
1	HBA_HUMAN	P69905	$0.83 \times 10^{-72}$	$100.0$	$100.0$
2	HBA_PANTR	P69907	$0.83 \times 10^{-72}$	$100.0$	$100.0$
3	HBA_PANPA	P69906	$0.83 \times 10^{-72}$	$100.0$	$100.0$
4	HBA_GORGO	P01923	$0.64 \times 10^{-71}$	$99.3$	$99.3$
5	HBA1_HYLLA	Q9TS35	$0.11 \times 10^{-70}$	$98.6$	$100.0$
6	HBA_PONPY	P06635	$0.29 \times 10^{-70}$	$97.9$	$100.0$
7	HBA_SEMEN	P01924	$0.81 \times 10^{-70}$	$97.9$	$99.3$
8	HBA_ATEGE	P67817	$0.17 \times 10^{-69}$	$96.5$	$100.0$
9	HBA_MACFU	P63107	$0.17 \times 10^{-69}$	$97.2$	$100.0$
10	HBA_MACMU	P63108	$0.17 \times 10^{-69}$	$97.2$	$100.0$

For demonstration purposes we applied a selection of the methods described above for the sequence and structure analysis of human hemoglobin. The corresponding source code is found as Jupyter notebooks in Additional file 1 and at https://github.com/biotite-dev/article-notebooks. Alternatively, the raw *Python* source code is available in Additional file 2. Examples for other *Biotite* functionalities can be viewed in the example gallery on the *Biotite* documentation website.

#### Identification of homologous sequences

To exemplify the usage of modular alignment search toolkit, we created a workflow to find homologs of the human hemoglobin $\alpha$-subunit ($\alpha$-globin) in the curated *Swiss-Prot* dataset from *UniProtKB* [9].

First the dataset was downloaded as FASTA file and its sequences along with the corresponding *UniProt* accessions and gene names were extracted. Sequences that belong to uncharacterized proteins or have viral origin were excluded. Furthermore the human $\alpha$-globin sequence was fetched (UniProt: P69905) as query sequence. To find homologous sequences of human $\alpha$-globin in a quick manner, a *k*-mer based alignment search was conducted. The spaced 6-mers of the *Swiss-Prot* sequences were indexed into a KmerTable. Repeat masking was omitted, to decrease the computation time for the sake of easy and fast reproducibility on commodity hardware. The spacing pattern was adapted from *MMseqs2* [14]. Then matches between the $\alpha$-globin sequence and the KmerTable were computed. To increase alignment search sensitivity, a similarity score threshold based on *BLOSUM62* [66] was used for matching. In order to filter the most promising matches, a double-hit strategy was implemented [17]: Only *Swiss-Prot* sequences with at least two matches on the same diagonal were considered in the downstream alignment search stages.

At the match position of each of the remaining hits an ungapped alignment with *X*-drop [12] criterion was performed (align_local_ungapped()). Hits, where the ungapped alignments exceeded a given threshold score, were subjected to gapped alignment within a band (align_banded()). *BLOSUM62* was used as substitution matrix and the gap penalty was taken from the *MMseqs2* default. The gapped alignments were sorted by their E-value, computed using an EValueEstimator and reported (Table 1).

#### Sequence conservation

**Fig. 4:**
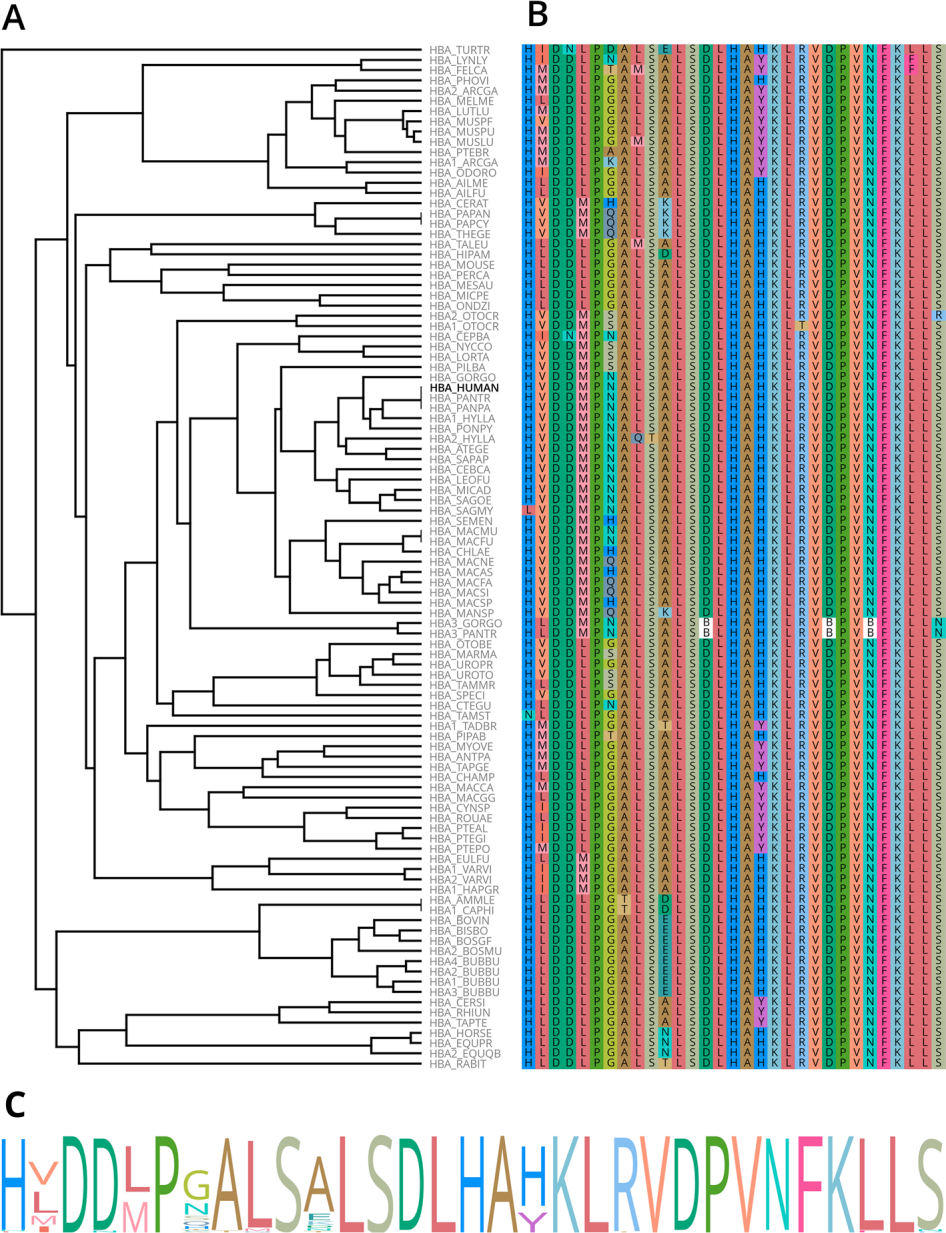
**MSA of** $\alpha$**-globin variants.** The MSA comprises the 100 identified sequences with highest similarity to the human variant. **A** Guide tree for the MSA. The human variant is highlighted. **B** MSA in vicinity to iron-binding histidine residue. The order of sequences is the same as in the tree. **C** Sequence logo in vicinity to iron-binding histidine residue, as highlighted in gray

Based on the identified homologs, the sequence conservation in the region of the iron-binding histidine residue was explored. For this purpose a MSA of $\alpha$-globin variants was conducted: From the identified homologs, the 100 sequences with highest similarity to human $\alpha$-globin were input to align_mulitple(). The guide tree of the MSA is shown in (Fig. 4A). The alignment was truncated to a certain number of amino acids around the iron-binding residue and displayed using the *flower* color scheme created with *Gecos* (Fig. 4B). Furthermore a SequenceProfile was created from the MSA in this region and used to create a sequence logo for more simple visual analysis of sequence conservation (Fig. 4C).

As the visualization functionalities of *Biotite* use *Matplotlib* [67], the elements of a figure can be easily customized using existing functionality from *Matplotlib*. In this case the subplot layout as well as the label highlighting was achieved completely using few *Python* statements. Since *Matplotlib* allows the user to access every graphical element, every aspect of a figure can in theory be customized.

To reduce the redundancy in the sequence selection, a clustered sequence dataset such as *UniRef* [68], could have been alternatively used as foundation. However, the *UniRef* datasets are orders of magnitude larger than *Swiss-Prot*. Thus for the purpose of this demonstration a smaller dataset was chosen.

#### Structure loading

**Fig. 5:**
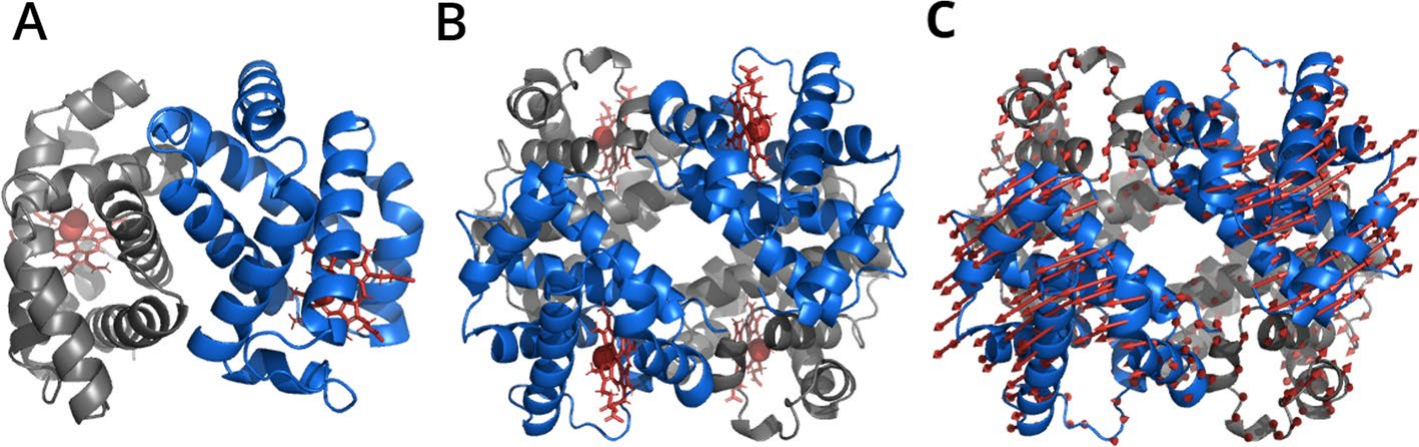
**Asymmetric unit and macromolecular assembly.** The atom coordinates and annotations are taken from PDB 6BB5. **A** Asymmetric unit of X-ray crystal structure. **B** Macromolecular assembly describing the functional tetramer of hemoglobin. **C** Normal mode with largest amplitude. The arrows depict the atom movement in this mode. The absolute arrow length is arbitrary

For the second part of the hemoglobin analysis a structure of human hemoglobin was chosen (PDB: 6BB5). The structure model was fetched from the PDB in *PDBx/mmCIF* format. The plain coordinates of the PDB entry correspond to the asymmetric unit of the X-ray crystal structure, representing a heterodimer (Fig. 5A). The functional hemoglobin tetramer, containing two $\alpha$-globin and two $\beta$-globin chains, was computed from transformations described by the structure file with get_assembly() (Fig. 5B).

#### Normal mode analysis

The normal modes of the tetramer were analyzed by means of an ANM using *Springcraft*. For this purpose the C$_{\alpha }$ atoms were selected from the assembly. The selected atoms as well as a residue type dependent ForceField (sdENM) [59] were used to create an ANM instance. The eigenvectors were computed from the ANM. The first relevant mode is shown in Fig. 5C.

#### Molecular docking

Next, molecular docking with *AutoDock Vina* [34] was used to find putative binding modes, so called poses, of heme in $\alpha$-globin. In case of the structure model at hand, the conformation of the ligand heme is already resolved. However, for the purpose of this example it was assumed that it is unknown, particularly as this docking protocol can be easily adapted to predict binding poses in other cases.

**Fig. 6:**
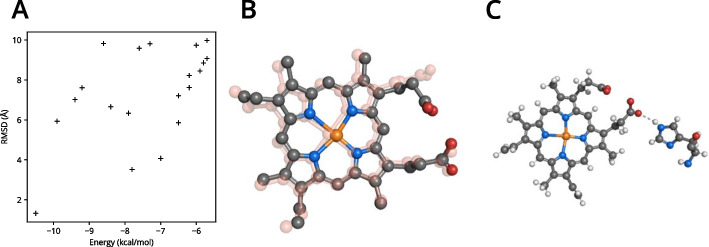
**Molecular docking of heme to** $\alpha$-**globin.** **A** Predicted binding energy and RMSD to experimentally determined conformation of heme binding poses suggested by *AutoDock Vina*. **B** The structure of the lowest energy binding pose. The experimentally determined conformation is shown in transparent red for comparison. **C** The lowest energy binding pose after hydrogen addition. The measured hydrogen bond to the protein is shown as dashed line

The structure model of heme was obtained from the CCD [33] and docked to $\alpha$-globin in vicinity of the binding pocket via the interface to *AutoDock Vina*. Since the correct binding mode is known, the suggested poses were assessed in terms of the root-mean-square deviation (RMSD) between the respective pose and the binding mode from the crystal structure (Fig. 6A). As assumed, the pose with the lowest predicted binding energy is also the one with the least RMSD (Fig. 6B).

*AutoDock Vina* does not include nonpolar hydrogen atoms in the calculated binding poses and polar hydrogen atoms are in an arbitrary orientation. To obtain a complete structural model nevertheless, the hydrogen atom positions for heme were predicted with *Hydride*. Eventually, hydrogen bonds between heme and $\alpha$-globin were identified with hbond() (Fig. 6C).

### Computational performance

**Fig. 7:**
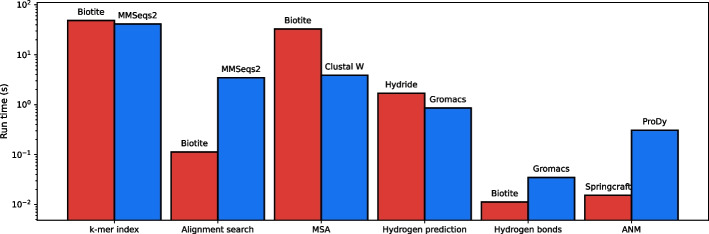
**Computational performance for different tasks.** *Biotite* and its extension packages are compared to other software in terms of computation time for selected tasks. The respective software is given on top of each bar. Each task was run 100 times and the average was taken, if not specified otherwise. *k-mer index*: KmerTable instantiation vs. mmseqs createindex for *Swiss-Prot* dataset. Repeat masking was omitted. Computations were performed using a single thread. Due to the high run time, this task was run only once. *Alignment search*: The workflow from ‘*Identification of homologous sequences*’ vs. mmseqs easy-search. *k*-mer indexing was not included in the time measurement. Computations were performed using a single thread. Instead of running mmseqs easy-search multiple times, it was run once with the according number of query sequence copies, to get a more realistic application scenario. *MSA:* align_multiple() versus clustalw -align [69] for 200 sequences from *SCOP* [70] globin family. Calculation of pairwise sequence distances and the guide tree is included. The task was run ten times. *Hydrogen prediction:* *Hydride* add_hydrogen() and relax_hydrogen() vs. gmx pdb2gmx [71] for a hemoglobin tetramer. *Hydrogen bonds:* hbond() vs. gmx hbond for a hemoglobin tetramer. *ANM*: Hessian calculation of an ANM in *Springcraft* vs. *ProDy* [72] for a hemoglobin tetramer

As *Biotite* aims to solve common questions in bioinformatics, also dedicated programs for many of these problems have been invented, often using the same or similar algorithm as *Biotite*. In order to be a flexible alternative to these programs, *Biotite* requires at least a similar computational performance to fulfill the respective tasks. If *Biotite* would require orders of magnitude larger computation time, its application would not be feasible in many cases.

In order to assess the performance, different tasks were chosen and computed with both, *Biotite* and one other representative software for the respective task. In case of *Python* libraries, including *Biotite* and its extension packages, file input and output was not included, to simulate the situation that multiple tasks would be performed in the same script without intermediate files. These benchmarks were conducted on a *Intel® Core™ i7-8565U* CPU (1.80 GHz). The *Snakemake* [73] workflow for the benchmarks is deposited in Additional file 3. The measured run times are shown in Fig. 7. Running the same benchmarks on different hardware showed similar trends (Additional file 4).

Note that in case of alignment search, MSA computation and hydrogen prediction, the compared implementations use different methods for the same problem and hence comparability is limited in terms of computation time and output of the software. Still, the benchmarks show that *Biotite* and its extension packages exhibit a computational performance on a similar time scale as dedicated popular software for the respective task. Solely the MSA computation is an order of magnitude slower than via *Clustal W* [69]. Since, there is a range of MSA software available, that have both sufficient flexibility and a command line interface, *Biotite* focuses on seamlessly interfacing to them and only provides a simple fallback solution for cases where the use of such software is not applicable.

An advantage of a program library compared to standalone programs in terms of performance becomes evident in cases were the workflow comprises multiple methods with little run time. Chaining multiple programs for such a workflow requires file input and output at the start and end of each program. In addition to the system-dependent read/write operation time the internal data representation needs to be converted into the respective file format and back again, which can take a notable portion of run time, if the remaining task is relatively fast. In contrast, when using a program library, the data can be kept in memory for multiple workflow steps. The additional file input and output operations may be a reason for the slower computation time of *MMseqs2* and *Gromacs* for alignment search and hydrogen bond identification, respectively.

## Conclusion

*Biotite*’s flexibility can be harnessed to tackle a wide range of problems, without the need to write ‘*glue*’ code for communication between different programs. For most tasks the implementation in *Biotite* performs similar or is even faster than dedicated software.

For some of the implemented methods, the implementation of the original publication is not (freely) available anymore, installation is cumbersome on modern architecture and operating systems, or the method description was purely theoretic. Here *Biotite* offers a modern alternative to apply such methods to current biological questions, that can be easily installed using the *pip* and *Conda* package managers.

## Availability and requirements

**Project name:** Biotite. **Project home page:** http://www.biotite-python.org/. **Operating system(s):** Windows, OS X, Linux. **Programming language:** Python. **Other requirements:** At least Python 3.8 and the packages requests, numpy, msgpack and networkx need to be installed. For plotting purposes matplotlib and for molecular visualization PyMOL is additionally required. **License:** BSD 3-Clause. **Any restrictions to use by non-academics:** None

## Supplementary Information

**Additional file 1**. Application example Juypter notebooks.

**Additional file 2**. Application example Python scripts.

**Additional file 3**. Benchmark workflow.

**Additional file 4**. Supplementary benchmarks.

## Data Availability

The source code for *Biotite* and its extension packages is available at https://github.com/biotite-dev/. The distributions of *Biotite*, *Ammolite*, *Hydride* and *Springcraft* used for creating the examples and benchmarks are available as archive [74].

## Abbreviations

ANM: Anisotropic network model
API: Application programming interface
CCD: Chemical components dictionary
ENM: Elastic network model
GNM: Gaussian network model
MSA: Multiple sequence alignment
NMA: Normal mode analysis
PEOE: Partial equalization of orbital electronegativity
PDB: Protein data bank
PFM: Position frequency matrix
PPM: Position probability matrix
PWM: Probability weight matrix
RMSD: Root-mean-square deviation

## Footnotes

1 These libraries usually revolve around the *ndarrays* from *NumPy*, making costly computations feasible in realistic time.
