# Supplementary material for: Biotite: new tools for a versatile Python bioinformatics library
Source: BMC Bioinformatics. 2023 Jun 5;24:236. doi: 10.1186/s12859-023-05345-6 (PMC10243083; doi:10.1186/s12859-023-05345-6)
Supplement: Supplementary file 4 — Additional file 4. Supplementary benchmarks. [file 12859_2023_5345_MOESM4_ESM.pdf]

Supplementary benchmarks

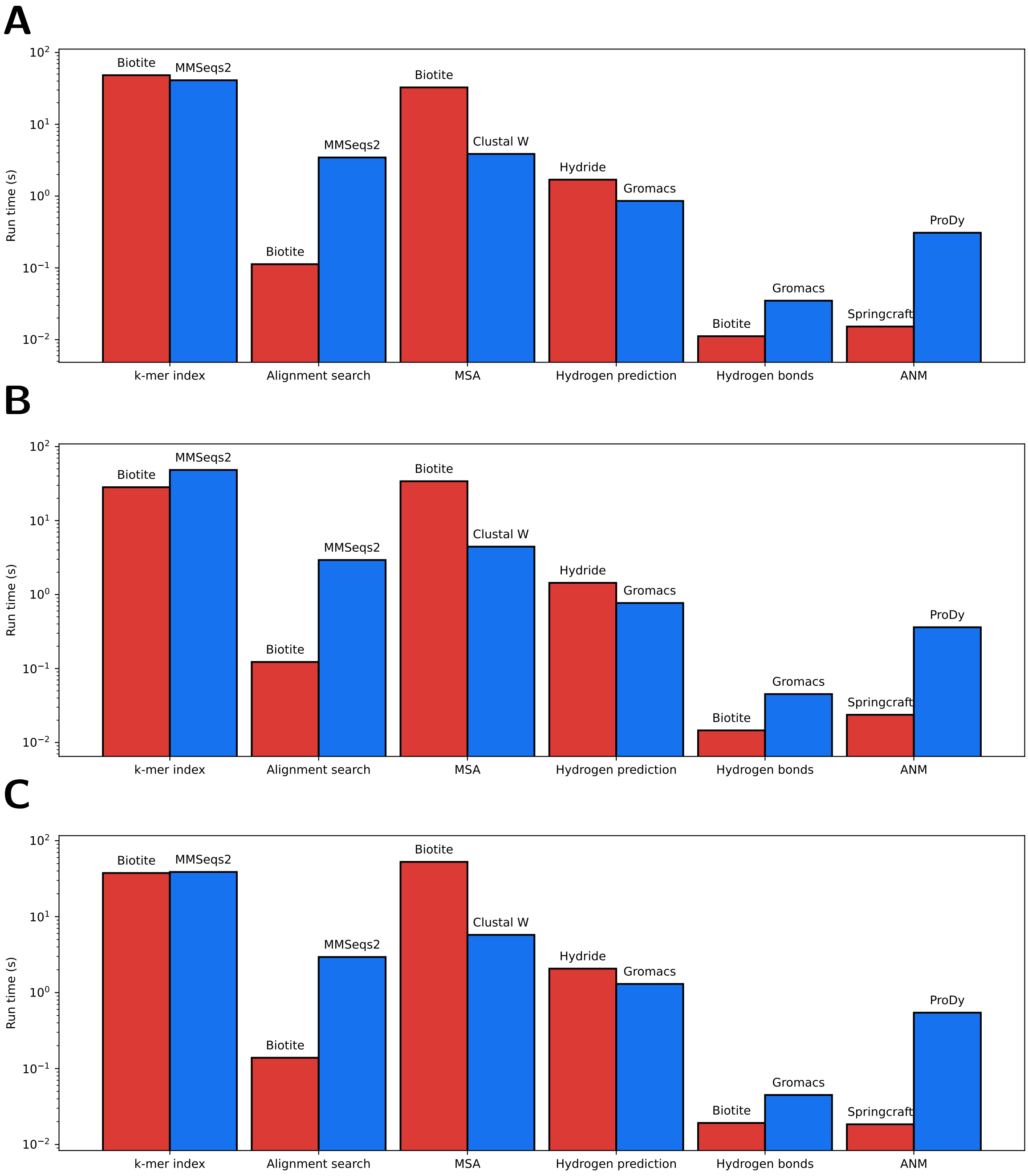

The shown benchmarks were conducted for the same tasks as described in Figure 7 of the main article on different machines.
A *Intel® Core™ i7-8565U* (1.8 GHz).
B *Intel® Xeon® Gold 6148 CPU* (2.4 GHz).
C *AMD Ryzen™ 7 1700X* (3.4 GHz).
